# Integration of RNA-Seq and proteomics data identifies glioblastoma multiforme surfaceome signature

**DOI:** 10.1186/s12885-021-08591-0

**Published:** 2021-07-23

**Authors:** Saiful Effendi Syafruddin, Wan Fahmi Wan Mohamad Nazarie, Nurshahirah Ashikin Moidu, Bee Hong Soon, M. Aiman Mohtar

**Affiliations:** 1 UKM Medical Molecular Biology Institute, Universiti Kebangsaan Malaysia, Bandar Tun Razak, Cheras, 56000 Kuala Lumpur, Malaysia; 2 Faculty of Science and Natural Resources, Universiti Malaysia Sabah, 88400 Kota Kinabalu, Sabah Malaysia; 3 Department of Surgery, Neurosurgery Division, Faculty of Medicine, Universiti Kebangsaan Malaysia, Bandar Tun Razak, Cheras, 56000 Kuala Lumpur, Malaysia

**Keywords:** Differentially expressed genes, Protein-protein interaction, Cell surface proteins, Network analysis, TCGA, GTEx

## Abstract

**Background:**

Glioblastoma multiforme (GBM) is a highly lethal, stage IV brain tumour with a prevalence of approximately 2 per 10,000 people globally. The cell surface proteins or surfaceome serve as information gateway in many oncogenic signalling pathways and are important in modulating cancer phenotypes. Dysregulation in surfaceome expression and activity have been shown to promote tumorigenesis. The expression of GBM surfaceome is a case in point; OMICS screening in a cell-based system identified that this sub-proteome is largely perturbed in GBM. Additionally, since these cell surface proteins have ‘direct’ access to drugs, they are appealing targets for cancer therapy. However, a comprehensive GBM surfaceome landscape has not been fully defined yet. Thus, this study aimed to define GBM-associated surfaceome genes and identify key cell-surface genes that could potentially be developed as novel GBM biomarkers for therapeutic purposes.

**Methods:**

We integrated the RNA-Seq data from TCGA GBM (*n* = 166) and GTEx normal brain cortex (*n* = 408) databases to identify the significantly dysregulated surfaceome in GBM. This was followed by an integrative analysis that combines transcriptomics, proteomics and protein-protein interaction network data to prioritize the high-confidence GBM surfaceome signature.

**Results:**

Of the 2381 significantly dysregulated genes in GBM, 395 genes were classified as surfaceome. Via the integrative analysis, we identified 6 high-confidence GBM molecular signature, HLA-DRA, CD44, SLC1A5, EGFR, ITGB2, PTPRJ, which were significantly upregulated in GBM. The expression of these genes was validated in an independent transcriptomics database, which confirmed their upregulated expression in GBM. Importantly, high expression of CD44, PTPRJ and HLA-DRA is significantly associated with poor disease-free survival. Last, using the Drugbank database, we identified several clinically-approved drugs targeting the GBM molecular signature suggesting potential drug repurposing.

**Conclusions:**

In summary, we identified and highlighted the key GBM surface-enriched repertoires that could be biologically relevant in supporting GBM pathogenesis. These genes could be further interrogated experimentally in future studies that could lead to efficient diagnostic/prognostic markers or potential treatment options for GBM.

**Supplementary Information:**

The online version contains supplementary material available at 10.1186/s12885-021-08591-0.

## Background

Glioblastoma multiforme (GBM) is the most common and lethal tumour of the central nervous system in adults [1]. Despite decades of efforts to tackle this disease, the median survival rate of GBM patients is still not improving [2]. GBM patients have an average life expectancy of 15 months post-diagnosis and the 5-years survival rate is less than 3% [3]. The standard-of-care GBM treatment generally consists of maximal safe surgical resection followed by radiotherapy and concomitant chemotherapy. However rapid post-treatment relapse and high intra-tumoral heterogeneity that could either arise naturally during disease progression or treatments-induced have made this disease intractable and more challenging to treat [4, 5]. Therefore, there is a pressing need for better and efficient diagnostic and therapeutic strategies for this disease.

Temozolomide, an orally administered DNA-alkylating drug, is the current and commonly used chemotherapy agent to treat GBM in the clinic [6]. This combination treatment of temozolomide and radiotherapy is referred to as the Stupp regimen and it is widely used as the standard-of-care for the treatment of GBM. The landmark study showed that the combination of radiotherapy and concomitant chemotherapy with temozolomide improve the patient’s prognosis compared to radiotherapy alone (median survival of 14.6 months vs 12.1 months, respectively) [6]. Alternative GBM treatment options such as the VEGF-targeting monoclonal antibody Bevacizumab, other DNA alkylating agents such as lomustine and carmustine implants, alternating electric field therapy and the checkpoint blockade inhibitor have thus far yielded low efficacy in treating GBM [2, 7, 8]. The Cancer Genome Atlas (TCGA) comprehensive GBM molecular characterizations have identified significant genetic alterations in several important oncogenic signalling pathways such as the RTK/Ras/PI3K (88%), p53 (87%) and pRB signalling pathways (78%) in GBM patients [9]. Several clinical trials are currently ongoing that aim to target these altered GBM oncogenic signalling pathways components using small molecule inhibitors and/or monoclonal antibodies. However, the results thus far were far from satisfactory [10]. This seems to suggest that instead of using a single agent targeting a specific component or pathway, novel treatments should consider the administration of several inhibitors targeting multiple different pathways.

The cell surface proteins or surfaceome serve as an information gateway that integrates and transduces extracellular cues into intracellular signalling cascades or vice versa. Surfaceome also play important role in cell adhesion and migration which are among the critical processes during tumorigenesis. Indeed, aberrant surfaceome expression and activity are frequently observed in many cancer types and therefore are good candidates for cancer diagnostic or biomarkers as well as therapeutic targets. Recent evidence has demonstrated that 56% of cell surface proteins are differentially expressed in GBM which are also present in cerebrospinal fluid or plasma, suggesting their potential use as biomarkers [11]. Of note, surfaceome expression is more dynamic than intracellular proteins and they could be sometimes cell type-specific [12, 13]. Mass spectrometry analysis showed that the average surfaceome size in brain cancer cell lines is higher than in other cancer types [12]. Thus, surfaceome genes in GBM may hold the key to understand GBM pathogenesis and drug responsiveness, in which targeting these genes may unravel potential ‘*druggable*’ stage in GBM pathways.

A comprehensive overview of the GBM surfaceome landscape has not been fully defined. Therefore, this study aimed to characterize the GBM surfaceome genes expression profile by unifying the two large RNA-Seq datasets from the TCGA (GBM) and GTEx (normal brain). We integrated and performed differential gene expression analysis on these two datasets because of the low number of normal brain tissue samples in the TCGA database. A previously annotated surfaceome gene set was employed to filter and identify the significant differentially expressed surfaceome genes in GBM. To further prioritize the high-confidence GBM cell surface signature, we integrated our transcriptomics analysis with GBM tissues and cell surface proteomics, and PPI hub gene analysis. Collectively, we identified a list of upregulated surfaceome genes in GBM that include *CD44, PTPRJ* and *HLA-DRA* in which their biological relevance in supporting GBM pathogenesis could be comprehensively investigated in future studies for the development of novel GBM diagnostic/prognostic or therapeutic strategies.

## Methods

### TCGA and GTEx data acquisition, normalization and quality control

The analysis combined the TCGA-GBM and GTEx normal brain RNA-Seq read count data. The GBM RNA-Seq gene raw read counts from TCGA were downloaded from Genomics Data Commons Data Portal (https://portal.gdc.cancer.gov). GTEx data were used for the normal brain tissues. The GTEx data used for the analyses described in this manuscript were obtained from the GTEx Portal on 29/03/19. We downloaded RNA-Seq gene raw read counts (from the cortex, frontal cortex, anterior cingulate cortex) from the GTEx portal (https://gtexportal.org/home/datasets). This allows us to perform the analysis of the differentially expressed gene on the 166 samples of GBM tumour from TCGA and 408 samples of normal brain tissues data from GTEx. The RNA-Seq raw read counts pre-processing steps involve are data filtering and data normalization. The normalization process of both data set was then performed by using mean as gene-level normalization using log_2_-counts per million where raw data are adjusted to account for factors that will prevent direct comparison of expression measures and to safeguard the expression distributions are similar for each sample across the whole experiment. Data that unlikely to be informative or simply erroneous data will be removed by using variance filter (less than 15) and low abundance (less than 4).

### Cell surface gene set classification and analysis

The identified differentially expressed genes (DEGs) of glioblastoma were classified into cell-surface genes set as discussed in the main text (See Results 2.4). The classification of the gene sets was performed based on the mapping set of DEGs with this resource. Other genes, which did not map to this resource were removed from the final dataset.

### Differential gene expression

DEGs analysis was performed using NetworkAnalyst [14], a web-based application tool for visualizing molecular and entity interactions. This platform utilizes the statistical method on data comparison from the R package, limma to identify genes whose expression is different. Genes that have adjusted *p*-value < 0.05 and log2 fold change |2| were considered as statistically significant DEGs.

### Functional annotation and pathway analysis

The enrichment analysis of the identified glioblastoma associated genes was performed using DAVID (https://david.ncifcrf.gov/), a web-based tool for analyzing functional gene analysis. The tool comprises databases from various public resources for biological analysis. The enrichment analysis such as GO and KEGG pathways were performed with top results as per gene counts.

### Identification of hub genes through PPI network analysis

A biological database for known and predicted protein-protein interactions called IMEx interactome database (https://www.imexconsortium.org) was used to construct the protein-protein interaction (PPI) of the DEGs. The network of interacting proteins was extracted and visualized using NetworkAnalyst. The top 87-gene modules of highly interacting gene clusters among the DEG were found with default parameters. For the classified gene sets, the PPI network was constructed and the network topological parameters i.e. degree and betweenness centrality were calculated.

### Co-expression network of CD44

Co-expression analysis was performed using Graphia Professional (https://kajeka.com/graphia-professional/), previously known as BioLayout *Express*^3D^ [15] using raw read counts and then saved as an “.expression” file. This contains a unique identifier for each row of data. Following import into Graphia Professional, a pairwise Pearson correlation matrix was calculated thereby performing a gene vs. gene comparison of the expression profile of each gene. All Pearson correlations where r > 0.7 were saved to a “.pearson” file. Based on a user-defined threshold of r > 0.75, an undirected network graph of the data was generated. In this context, nodes represent individual genes and the edges between them represent Pearson correlation coefficients above the selected threshold (r > 0.75). *CD44* was selected along with its neighbour in the network, representing *CD44* co-expression partners. The class set of *CD44* co-expressed genes were visualized to compare the expression values in this class set with genes in normal samples.

## Results

### Patients’ characteristics of TCGA and GTEx

We utilized the publicly available TCGA and GTEx RNA-Seq database as our primary sources of GBM tumour and normal brain tissue transcriptomic data, respectively. We downloaded the datasets containing RNA-Seq gene expression profiles and clinical information of 166 patients from TCGA-GBM and 408 normal brain tissues from the GTEx database. The combined data were stratified based on sex, age and treatment as shown in Table 1. Out of a total of 166 GBM cases, 104 cases (62.7%) were male, 56 cases (33.7%) were female and 6 cases did not have sex specification. GBM is more prevalent in patients aged *≥*60 years old which accounts for 42.8% of total cases in the TCGA GBM cohort. Fifty-two patients (31.3%) have undergone treatments whereas 62.1% of cases did not have any treatment data. Unfortunately, the clinical data for the GTEx normal brain samples are not publicly available.

**Table 1:** TCGA GBM patients’ clinical data

		Cases
**Sex**	Male	104 (62.7%)
	Female	56 (33.7%)
	Not reported	6 (3.6%)
	**Total**	**166**
**Age at diagnosis (Years old)**	< 40	9 (5.4%)
	40–60	49 (29.5%)
	> 60	71 (42.8%)
	Not reported	37 (22.3%)
	**Total**	**166**
**Treatment**	Yes	52 (31.3%)
	No	11 (6.6%)
	Not reported	103 (62%)
	**Total**	**166**

### Identification of differentially expressed genes in glioblastoma

**Fig. 1:**
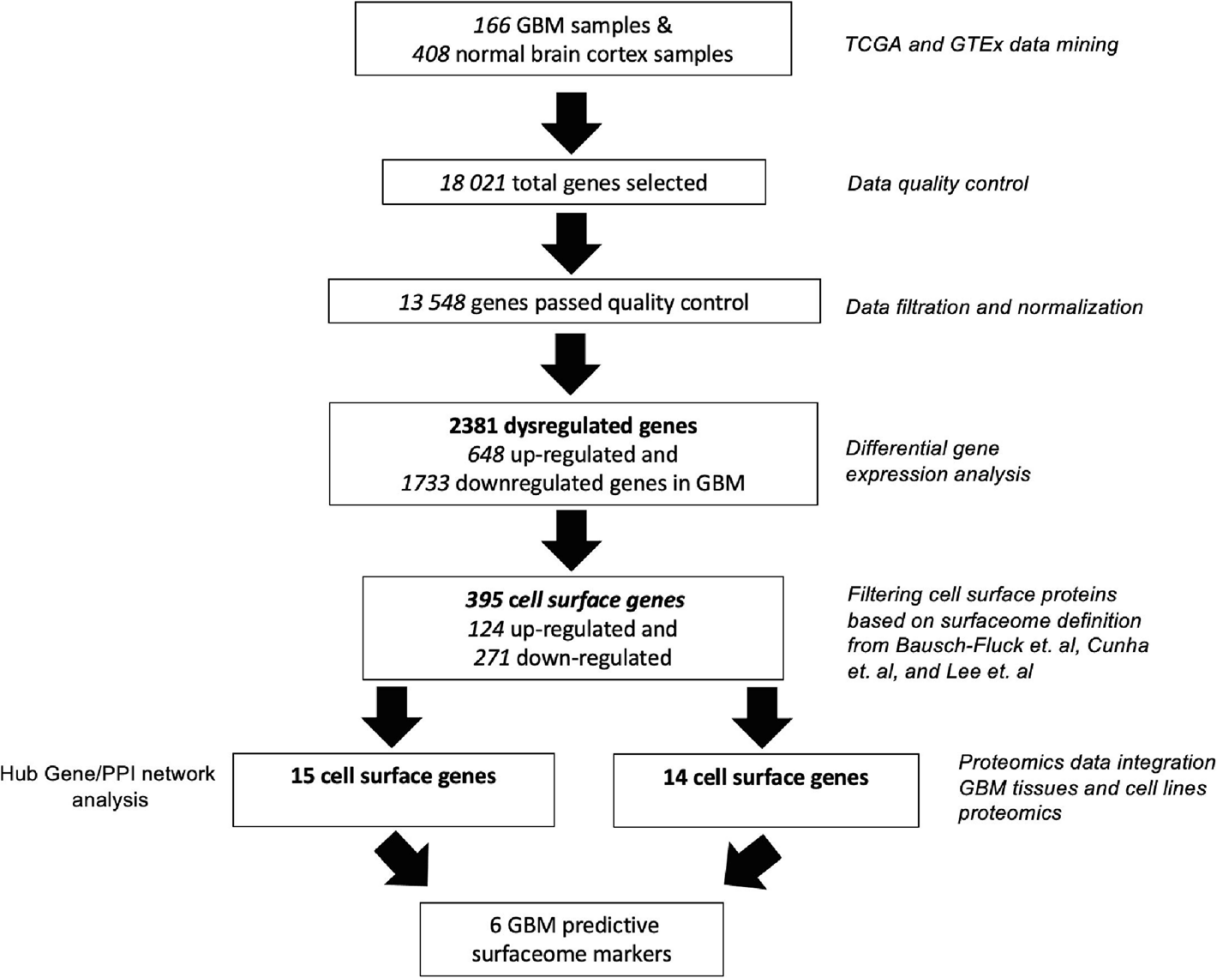
Analysis pipeline to obtain the GBM predictive surfaceome markers applied from the initial TCGA GBM and GTEx data integration

**Fig. 2:**
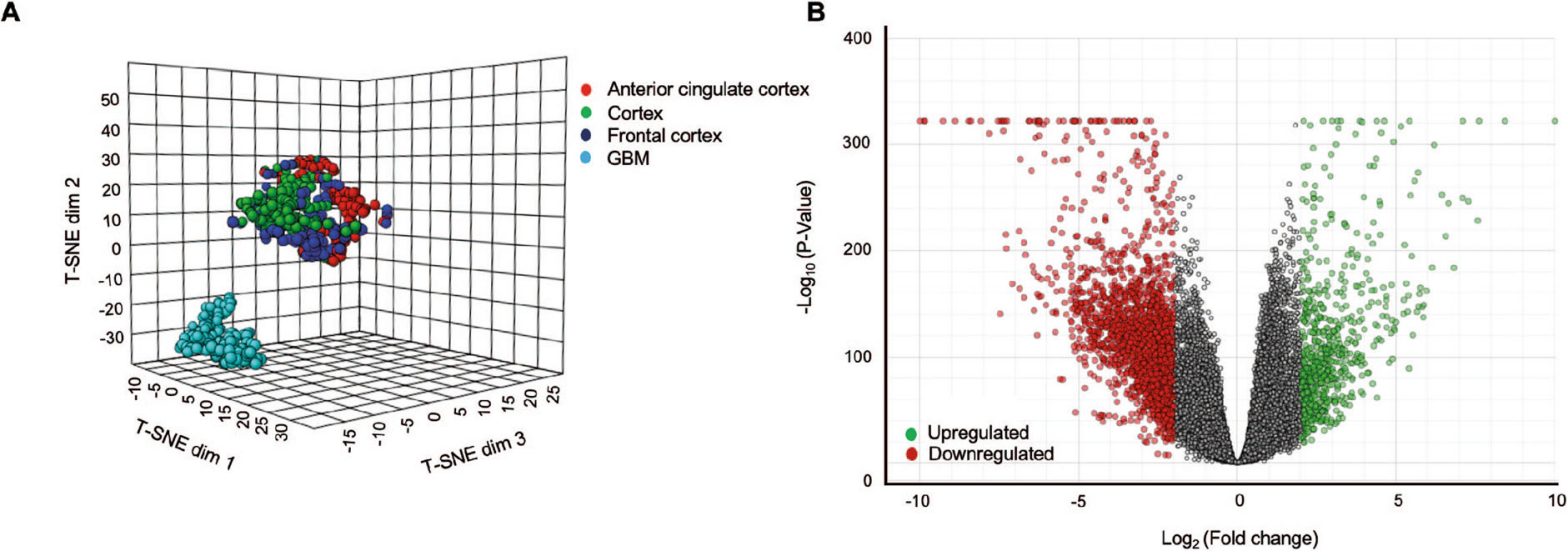
Identification of global differentially expressed genes in GBM. (A) t-SNE plots showing the GBM and GTEX data cluster. (B) Volcano plot of the differentially expressed genes in GBM versus normal brain tissues. Genes that are significantly dysregulated in GBM versus GTEx (log2 fold change |2|) were highlighted in red (downregulated) and green (upregulated)

The analysis pipeline employed in this study is depicted in Fig. 1. Briefly, the RNA-Seq raw read counts from the two large compendiums, TCGA and GTEx were utilized to identify the differentially expressed genes between GBM and normal brain tissues. Since most GBM cases are generally found in the supratentorial region of the brain such as the cerebral hemisphere [16], we only extracted the RNA-Seq profiles of this region namely the cortex, frontal cortex, anterior cingulate cortex as per GTEx description. We performed t-distributed stochastic neighbour embedding (t-SNE) analysis to reflect the directionality of transcripts expression among GBM tumour and normal brain tissues read count values. The t-SNE plot showed that all RNA-Seq profiles of all GTEx cortex regions clustered together while the GBM RNA-Seq profiles form a separate cluster, thus confirming distinct expression patterns between these groups (Fig. 2A). In total, RNA expression data from 18,021 genes were obtained from these combined TCGA and GTEx datasets but only 13,548 genes passed the quality control check. By applying the cut-off criteria log_2_ fold change |2| and adjusted *p*-value < 0.05, we identified 2381 genes as significantly differentially expressed genes (DEGs) in GBM, of which 648 genes were upregulated and 1733 genes were downregulated (Fig. 2B). The detailed information of the differential gene expression analysis is listed in Supplementary Table S1.

### Functional enrichment analysis and classification of DEGs

The significant DEGs were then subjected to functional enrichment analysis using Gene Ontology (GO) and Kyoto Encyclopedia of Genes and Genomes (KEGG) tools to define their properties and putative biological relevance in GBM. Interestingly, the GO cellular component analysis of both upregulated and downregulated DEGs showed enrichment of cell surface and membrane-associated proteins (Supplementary Fig. S1A and B). The KEGG pathway enrichment indicated that the upregulated DEGs are involved in pathways related to infectious diseases, pathways in cancer and cell adhesion (Supplementary Fig. S1C). Downregulated genes mainly involve in neuroactive ligand-receptor interaction and major cellular signalling pathways (Supplementary Fig. S1D).

### Identification of GBM cell-surface antigen candidates

The DEGs were then further filtered and classified into the surfaceome gene set as previously defined by Bausch-Fluck et al. [13], Cunha et al. [17] and Lee et al. [18]. These studies utilized different criteria and stringency in curating the surfaceome gene list. From the overall DEGs in GBM, we identified 395 common cell surface genes within these three surfaceome definitions, including 124 upregulated and 271 downregulated genes (Supplementary Fig. S2A and Supplementary Table S2). We further classified the surfaceome according to their main subclasses, which are receptors, transporters, enzymes, miscellaneous and unclassified, as previously reported by Almén et al. [19]. Among the defined surfaceome subclasses, 42.8% of the significant differentially expressed surfaceome in GBM belong to the receptor subclass (Supplementary Fig. S2B). KEGG analysis of the GBM-enriched cell surface proteins identified pathways related to immune defence and infectious disease pathways while GBM-deficient cell surface genes are enriched in pathways related to neuroactive ligand-receptor interaction and major cellular signalling pathways (Supplementary Fig. S3A and B). These findings are almost similar to the enrichment analysis of overall DEGs in GBM (Supplementary Fig. S1C and D) suggesting that surfaceome has significant roles in dictating GBM cellular activities.

### Identification of GBM cell-surface signature by integration of proteomics and transcriptomics data analysis

Thus far, we have (i) classified the overall DEGs in GBM using transcriptomics data and (ii) highlighted the differentially expressed cell-surface genes in GBM. Even though this transcriptomics analysis is very informative for biomarker discovery, we aimed to add another layer of analysis to select a more high-confidence cell surface signature for GBM. To attain this, we integrated our transcriptomics analysis data with the publicly available proteomics data. This integration will validate the cell surface genes prediction and eliminate the possible discrepancy between the expression levels of mRNAs and proteins due to post-transcriptional and post-translational modifications. Thus, we gathered the publicly available quantitative mass spectrometry analysis data for both GBM tissues and cell lines. We postulated that GBM tissues and cell lines might have different cell surface repertoires and therefore it is important to stratify between these two sources. Additionally, GBM cell lines cell surface signature, as identified in this present study, could be validated experimentally in future functional studies.

**Fig. 3:**
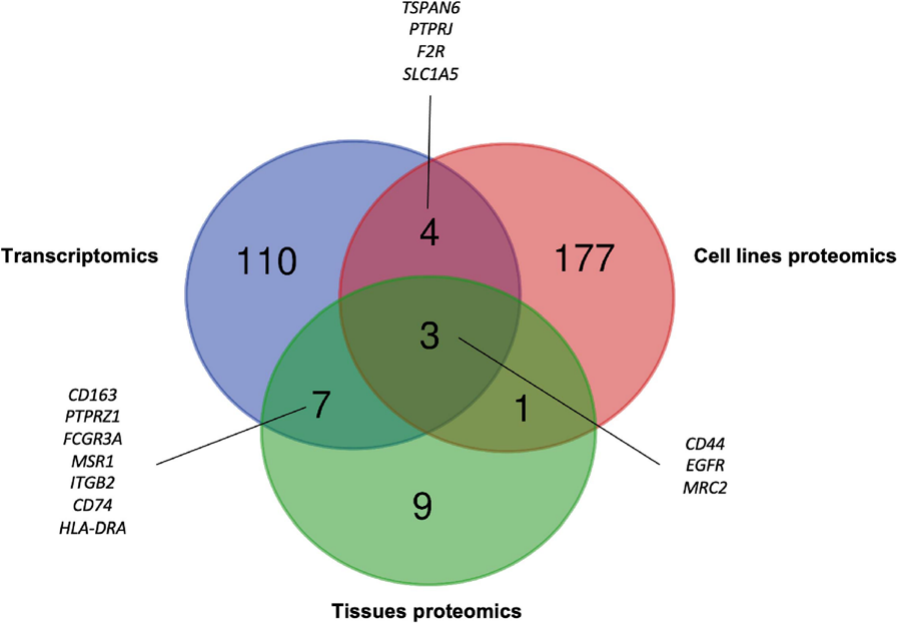
Integration of TCGA GBM transcriptomics, GBM tissues proteomics and cell lines proteomics data

Mass spectrometry analysis of five GBM cell lines revealed the upregulation of EGFR, CD44, PTPRJ, SLC1A5, F2R, and TSPAN6 proteins in these samples [12], whereby the expression level of these proteins was in concordance with our transcriptomics data analysis (Fig. 3). For tissue proteomics, we found several studies that performed comparative GBM vs. normal brain tissues proteome profiling [11, 20–23]. However, some of these studies either identified only a limited number of proteins or the data are not downloadable. Only one study by Polisetty et al. has identified a large number of proteins in their proteome profiling study that included 1834 high-confidence membrane proteins with more than 2-fold change [11]. We, therefore, used this dataset where we performed an integrative analysis with our analyzed transcriptomics data and identified 10 overlapped genes, *MRC2, FCGR3A, HLA-DRA, CD44, CD74, MSR1, CD163, EGFR, ITGB2, PTPRZ1* (Fig. 3). The mRNA expression levels correlated with the protein expression levels except for the *PTPRZ1* where the mRNA levels showed upregulation while proteomics data showed downregulation (Supplementary Table S3 and S4). In total, there are 14 genes from the combined tissues and cell lines proteomics that overlapped with our transcriptomics data (Fig. 3). It is important to note that proteins identification in mass spectrometry can be limiting due to protein isolation methods, proteins solubility, and other intrinsic variations that affect the proteins abundance as well as the sensitivity and detection capability of the MS instrumentation [24, 25]. Thus, these limitations may underestimate the results of transcriptomics prediction and proteomics discoveries.

### Surfaceome protein-protein interaction network cluster analysis and prioritization of high-confidence GBM cell surface markers

**Fig. 4:**
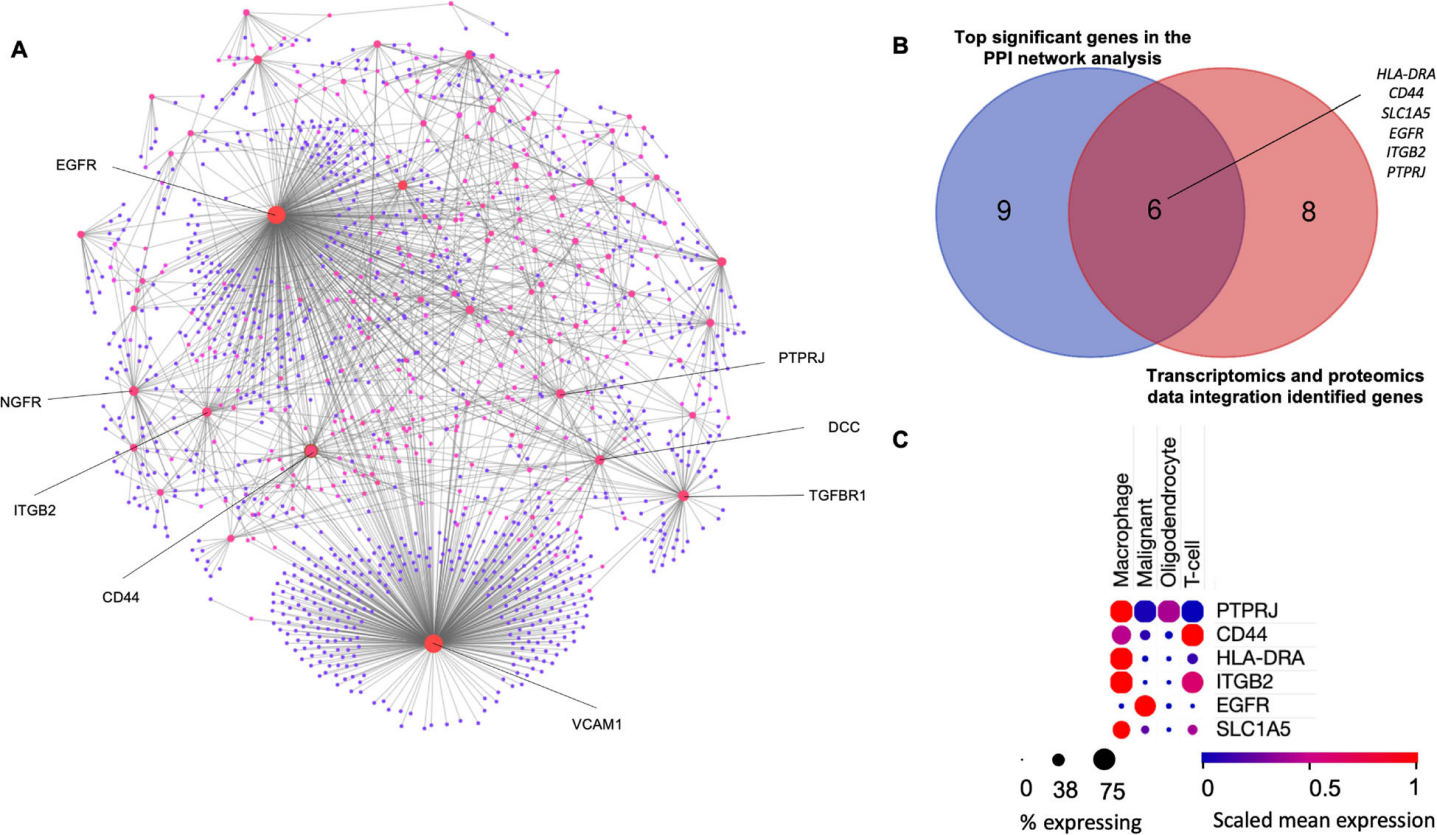
Prioritization of 6 high-confidence GBM surface marker genes. (A) Protein-protein interaction network analysis of the significantly upregulated GBM surfaceome genes. (B) Venn diagram showing the genes that are overlapped between the PPI network and transcriptomics-proteomics data integration analysis. (C) Expression of the 6 high confidence surface markers on the basis of GBM cell microenvironment extracted from scRNA-seq data [26]

We set out to further analyze the GBM-enriched cell surface markers using protein-protein interaction (PPI) network analysis. This is to better understand the interplay between the cell surface genes within the identified DEGs as well as with other genes. More importantly, this would enable us to further select the genes that are highly interconnected from the integrated proteomics and transcriptomics analysis. Network analysis of the identified differentially expressed cell surface protein genes was performed using NetworkAnalyst [14] to determine the relationship between genes according to the network topological parameters such as degree and betweenness. These parameters reflect the role and property of proteins within the network. The nodes and edges in the PPI network represent the proteins and their interactions, respectively. The GBM-enriched cell surface proteins network contains 1321 nodes and 1767 edges interactions based on a number of validated features including functional experiments, co-expression analysis, text mining, neighbourhood, gene fusion and databases (Fig. 4A). We identified 87-gene modules of clusters and the top cluster genes with more than 30 interactions include *VCAM1, EGFR, TGFBR1, CD44, NGFR, ITGB2, DCC, PTPRJ, ANBCA1, HLA-DRA, CCR5* and *CSF1R* (Fig. 4A and Supplementary Table S5). Vascular Cell Adhesion Molecule 1 (VCAM1) has the highest interacting cluster as it was found to have 426 degrees with a 422,712.18 betweenness score. We subsequently mapped the 14 genes identified from the integrated transcriptomics and proteomics data analysis (Fig. 3) with the top genes that have at least 20 interactions from the PPI network analysis. We found 6 genes that were in common between these two datasets which represent the high-confidence GBM predictive surfaceome markers (Fig. 4B). It is important to highlight that our analysis thus far integrated multi-OMICS data from bulk samples. It is known that GBM suffers from inter and intra-tumoural heterogeneities that contribute to the emergence of several molecular subtypes [26–28]. Single-cell RNA-sequencing (scRNA-seq) corroborated that the co-existence and interaction between different cells population within the GBM microenvironment drive the GBM cells pro-oncogenic cellular programs. To examine this, we integrated our analysis with the scRNA-seq data containing 24,131 cells from adult and pediatric GBM patients [26]. The study further stratified the cells within the GBM microenvironment into macrophages, oligodendrocytes, T-cells and malignant cells (Supplementary Fig. S4). Of the identified 6 high-confidence cell surface markers, only EGFR was strongly expressed in the malignant GBM cells, while the other genes were strongly expressed in the macrophages (Fig. 4C).

### Validation of high-confidence GBM signature gene and survival-expression correlation analysis

Next, we validated the expression profiles of the identified 6 high-confidence cell surface markers using an independent database, Gene Expression Profiling Interactive Analysis (GEPIA) [29]. GEPIA also combines the TCGA and GTEx gene expression data that were processed from raw reads count and unified using its own pipeline. In line with our findings, the identified GBM cell surface signature genes were confirmed to be significantly upregulated in the GBM GEPIA database (Supplementary Fig. S5A – F). To investigate whether the expression level of these signature genes would modulate/influence GBM patients’ prognosis, we first performed the overall survival analyses on GBM patients who had high or low expression of each of these 6 genes (Supplementary Fig. S6A – F).

However, there were no significant differences in the overall survival between patients who had high or low expression of these 6 individual prioritized genes. Since GBM patients have a low overall survival rate (average < 2 years’ survival post-diagnosis), we postulated that it would be more appropriate to look at the disease-free survival endpoint rather than the overall survival. Moreover, the overall survival endpoint is more suited for a longer follow-up period (typically 5 years) for the data to be meaningful [30]. Hence, we examined the disease-free survival profile of the GBM patients in a similar fashion. We found that high expression of *CD44*, *PTPRJ* and *HLA-DRA* were significantly correlated (*p* < 0.05) with poor disease-free survival in GBM patients (Supplementary Fig. S7A – S7F).

In addition to performing survival analysis on the individual gene, we also assessed whether combining the level of all 6 GBM signature genes as a group could predict the GBM patients’ overall survival and disease-free survival. We observed that there was no statistically significant difference in the overall survival and disease-free survival between patients who had high expression and low expression of the signature group (Supplementary Fig. S8A – B). Interestingly, by combining only *CD44*, *PTPRJ* and *HLA-DRA* in the gene signature, we found that subjects with high expression of this signature group had significantly poor disease-free survival (*p* < 0.0084) compared to patients who had low expression of these genes (Supplementary Fig. S9). However, there was still no significant difference in the overall survival between GBM patients in this signature group (Supplementary Fig. S9).

### Co-expression network of CD44

**Fig. 5:**
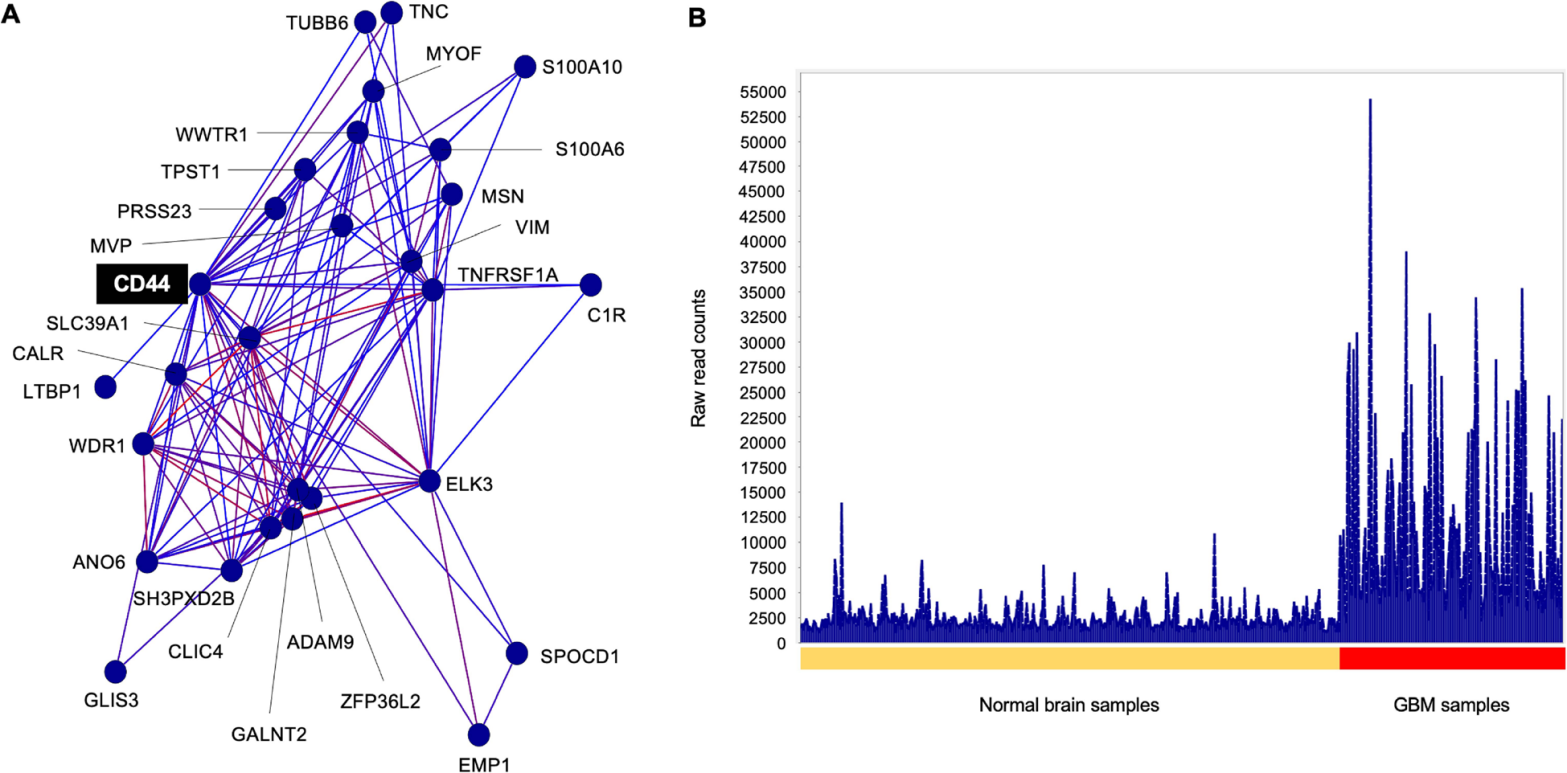
CD44 gene co-expressed network analysis. (A) CD44 gene co-expressed network with Pearson correlation value, r > 0.75. Nodes represent genes and edges are coloured on a sliding scale according to the strength of the correlation (red, *r* = 1.0 and blue, *r* = 0.75). (B) Histograms of CD44 co-expression cluster from (A) showing the average expression of genes on GBM tumour (red bar) and normal (yellow bar)

CD44 is a transmembrane receptor and has multifaceted functions in both normal and disease physiology. OMICS studies have identified CD44 to be overexpressed in many types of cancer including glioblastoma [31, 32]. Based on our analysis, CD44 seems particularly important as it can be both identified in transcriptomics and proteomics-based approaches, among the top hub gene and whose high expression correlated with poor disease-free survival. In addition, scRNA-seq identified CD44 was enriched in mesenchymal-like cell state in GBM population, and orthotopic xenografts of these CD44-enriched fractions in immunocompromised mice was able to initiate GBM [26]. We performed a co-expression network analysis to further interrogate its association with other genes using our transcriptomics. The nodes represent in the network analysis represent genes, while the edges represent Pearson correlation above r > 0.75. The neighbouring genes connected to CD44 was extracted and shown in Fig. 5A. There are 27 genes in this complex connected to CD44. Among the highly correlated genes are ELK3, CLIC4, GALNT2, TNC, and VIM. All genes in this CD44 co-expression cluster are highly expressed in GBM compared to normal brain samples (Fig. 5B), further corroborating the biological relevance of CD44 in supporting GBM pathogenesis.

### Identification of drugs targeting GBM signature and CD44 network

We next determined whether there are any clinically approved drugs targeting the identified high confidence GBM cell surface markers (Supplementary Fig. S4) and components of the constructed CD44 co-expression network (Fig. 5A). To achieve this objective, we utilized the Drugbank database (https://go.drugbank.com/) and our searches yielded several approved drugs that can be potentially effective or repurposed to target CD44, EGFR, C1R, CALR and TNFSR1A (Table 2). Hyaluronic acid, for example, is a clinically approved ligand for CD44 and this drug has been administered in the clinic to treat diseases such as osteoarthritis [33]. Excessive hyaluronic acid administration has been demonstrated to inhibit tumour growth, possibly by impeding cell-cell interaction [34]. Besides, the use of nanomaterials to enhance the efficiency of hyaluronic acid delivery for cancer therapy is also actively being explored [35, 36]. Thus, the promising features of hyaluronic acid in mediating enhanced drugs or genes delivery to cancer cells via the overexpressed CD44 receptor could potentially be applied and developed for novel GBM therapeutic strategies. In regards to EGFR, several inhibitors and monoclonal antibodies have already been therapeutically approved to target this protein due to its roles as an important driver of tumorigenesis in many cancer types [37].

**Table 2:** Available approved drugs to target the identified GBM molecular signature and CD44 co-expression network

Gene	Approved drugs
CD44	Hyaluronic acid
EGFR	Cetuximab, Gefitinib, Erlotinib, Lapatinib, Panitumumab, Lidocaine, Vandetanib, Afatinib, Necitumumab, Osimertinib, Neratinib, Brigatinib, Foreskin keratinocyte, Fostamatinib, Dacomitinib, Zanubrutinib
C1R	Palivizumab, Conestat alfa, Human C1-esterase inhibitor
CALR	Tenecteplase, Antihemophilic factor, Melatonin, Lonoctocog alfa, Moroctocog alfa
TNFSR1A	Tasonermin

Moreover, of the 28 components of the CD44 co-expression network (Fig. 5A), only C1R, CALR, and TNFSR1A have drugs that can modulate them (Table 2). For instance, 3 drugs can be used or repurposed to target C1R. The pharmacological activity of Palivizumab to bind the C1R subcomponent is under investigation, whereas the conestat alfa and human C1-esterase inhibitor can directly target the C1R subcomponent and disrupt the complement system activation.

## Discussion

The surfaceome comprise cellular frontiers that permit/inhibit signal transduction as well as playing important roles in modulating cells proliferation, migration and invasion, and cells-cells interaction. The surfaceome can organize itself at a nanoscale resolution [38]. This spatiotemporal nanoscale organization could define the cell identity and phenotypes, and capacity to communicate with microenvironments such as the extracellular matrix, growth factors, hormones and drugs. Due to their accessibility on the cell membrane, surfaceome proteins are ideal candidates for biomarkers and often targeted for drugs development. Over 50% of drugs curated in the DrugBank target the surfaceome. In addition to their ubiquitous expression on the plasma membrane, the extracellular stalks of these cell surface proteins can be cleaved and released into the bloodstream, making them suitable targets for blood-based diagnostics. Surfaceome can also be draped with glycans during post-translational modifications, which will mediate their interaction with other proteins that reside on either the same or neighbouring cells as well as with the microenvironments [38]*.* Dysregulated surfaceome expressions and functions have been shown to promote tumour formation and progression [39]. Therefore, scientists have begun profiling and cataloguing surfaceome in various types of cancers [40–43]. These cell surface proteins can be elevated in cancer cells in which they can respond to the increased level of growth factors, rendering cancer cells to sustain their infinite proliferative capabilities [44] and interact with the microenvironment that could either directly or indirectly modulate the tumour growth and metastatic capabilities [45].

The GBM transcriptomics dataset has been previously utilized to uncover genes that support GBM pathogenesis as well as genes that have potential prognostic values [46–48]. For example, Nicolasjilwan et al. analyzed the TCGA database to predict the survival of GBM patients based on clinical features, MRI images genomics alterations [46]. However, most TCGA GBM differential genes expression analyses either relied on a low number of normal brain tissue samples, in which the TCGA GBM cohort contained only 5 normal brain tissues RNA-Seq data, or the data were combined with the GBM TCGA microarray data. This might create an imbalance that would lead to inaccuracy or bias in the downstream analysis. Hence, to increase the robustness of this study in identifying the significantly upregulated GBM surfaceome repertoire, we included the normal brain tissues GTEx RNA-Seq database TCGA in our analysis. On a similar scale, the GTEx studies have performed genes expression profiling in more than 11,000 samples across multiple human tissues from nearly 1000 healthy donors. We compared the TCGA GBM and normal cortex GTEx RNA-seq data and identified 2381 significant differentially expressed genes in GBM, in which 648 were upregulated and 1733 downregulated genes. In agreement with the previous GBM proteomics profiling study [12], the GO cellular compartment analysis showed that most of the dysregulated genes in GBM encode for the cell surface proteins, suggesting the importance of cell surface proteins in GBM pathogenesis.

Of the 2381 significant DEGs in GBM, 395 genes encode for cell surface proteins, in which 124 and 271 genes were found to be significantly upregulated and downregulated, respectively. Interestingly, receptor subclass was the predominant dysregulated genes in GBM, suggesting the crucial roles of cell surface receptors in supporting GBM pathogenesis. This was indeed in line with several studies reporting the implications of cell surface receptors dysregulation in the pathogenesis of many cancer types [49]. For this reason, the development of cancer treatment strategies has been revolved around targeting the cell surface receptors such as the receptor tyrosine kinases (RTKs) [50] and G protein-coupled receptors (GPCRs) [51]. Therefore, targeting the cell surface proteins particularly the receptor subclass could potentially be further explored as novel GBM therapeutic options.

Robust cancer biomarkers are those that could be reproducibly identified by multi-omics platforms or reported in several different studies. To this end, we integrated the analyzed transcriptomics data with publicly available GBM proteomics data to prioritize high-confidence cell surface proteins. Also, due to post-transcriptional and post-translational modifications, the mRNAs expression level is sometimes not correlated with their respective protein expression levels [52]. After mapping the prioritized genes from the transcriptomics-proteomics integrative analysis with the PPI network analysis data, we identified 6 genes; *HLA-DRA, CD44, SLC1A5, EGFR, ITGB2, PTPRJ*, whereby we considered these genes as the high-confidence GBM predictive surface markers. Previously integrated transcriptomics based on bulk expression profiles suggested that GBM is heterogeneous and can be clustered into at least three subtypes namely pro-neural, classical; and mesenchymal [28]. Recently, scRNA-seq analysis confirmed the intra-tumoural heterogeneity of GBM in which it can exist in multiple states with distinct cells and transcriptional programs that can be dynamically transitioned into different subtypes [27]. Most of the identified 6-gene signature belongs to macrophage cell type while only EGFR is specific to GBM (Fig. 4C and Supplementary Fig. S4). Although most of the hits are not GBM-specific genes, these different cells are part of the GBM microenvironment or tumour niche, which are equivalently important in driving GBM pathogenesis. Hence, regulation of these genes within the tumour microenvironment recapitulates the cellular program, plasticity and genetic drivers of GBM. Overall survival analyses revealed that there was no significant difference in the overall survival between patients who had high and low expression of these 6 genes, either the genes were analyzed individually or when combined. However, when looking at the disease-free survival, patients who had high expression of *CD44, PTPRJ,* and *HLA-*DRA, either individually or as a group, had significantly poor disease-free survival (Supplementary Fig. 6 and 8B) compared to subjects with low expression of the genes. These findings indicate that these 3 genes, *CD44, PTPRJ,* and *HLA-DRA,* could potentially be developed as GBM prognostic markers in the clinic.

In addition to identifying the already known GBM drivers like CD44 and EGFR, our integrative analysis approach has also enabled us to identify potential novel genes that have not either been reported or thoroughly discussed in the context of GBM. For instance, within the 6 GBM signature genes, ITGB2 has not been widely associated with the pathogenesis of GBM. ITGB2 encodes for cell surface protein that is important in regulating cell adhesion and cell-surface mediated signalling [53]. Hence overexpression of this protein is relevant in promoting cancer growth possibly by modulating cancer cells adhesive and migratory properties, and the pro-oncogenic signalling cascades. Though there are *in-silico* and in-vitro studies that associated the ITGB2 as one of the important genes in cancer, the exact mechanisms of how this gene promotes GBM remains elusive and worth to be investigated in the future [11, 54, 55]. Human leukocyte antigen (HLA)-DRA is a classical major histocompatibility complex (MHC) class II molecule that plays important role in immune responses modulation. High expression of the HLA-DR gene family has been associated with more aggressive tumour grade in gliomas and poor prognosis [56, 57]. Nonetheless, the functions of HLA-DRA in driving GBM growth has not been fully elucidated.

PTPRJ gene is a member of the protein tyrosine phosphatase (PTP) family whose substrates include the RTKs such VEGFR, PDGFR and EGFR [58]. Since the RTKs pro-oncogenic properties are well-established in which their activation largely depends on phosphorylation, PTPRJ is thus deemed to function as tumour suppressor proteins due to its function as a phosphatase that can negatively regulate the signalling pathway. This was also evidenced by the ectopic expression of PTPRJ in in-vitro models that resulted in cell growth inhibition [59, 60]. In contrast to these previous reports, we found that *PTPRJ* expression was upregulated in GBM and led us to suggest that PTPRJ might have a pro-oncogenic role in GBM pathogenesis. To our knowledge, there have been no previous reports linking *PTPRJ* expression and function with GBM pathogenesis. This notion of PTPRJ potential ‘double-edged sword’ and GBM-specific pro-oncogenic function needs to be investigated further. SLC1A5, another hit target from our analysis, is a neutral amino acid transporter in which its high expression has been implicated in many cancer types including GBM [61]. In GBM, SLC1A5 expression is under the control of pro-oncogenic c-Myc protein but how this transporter supports the tumour cells proliferation and growth remain poorly understood [62].

As highlighted above, the identification of CD44 and EGFR in this present study is expected because they have been previously described as one of the key targets for GBM [32, 63]. This validates the robustness of our approach in the sense that not only our analysis identified several novel genes, but also the findings overlap with previous studies. Since EGFR pro-oncogenic roles have been widely implicated in many cancer types and several drugs have been developed and clinically approved to target EGFR [37, 64, 65], we focused our analysis on CD44. The CD44 encodes for transmembrane glycoprotein that serves as the receptor for hyaluronic acid, a component of the extracellular matrix, and several other ligands including osteopontin, fibronectin and collagen [32]. The CD44 antigen has been implicated in modulating tumorigenesis in many cancer types in which high expression of this CD44 increases cancer cells proliferation, motility and survival as well as promoting cancer metastasis [66]. In GBM, high expression of CD44 was identified in the proteogenomic profiling of GBM tissues [23] and further classified as a GBM cell surface antigen in a systematic analysis [31]. Interestingly, this transmembrane glycoprotein can be cleaved and secreted into the vasculatures, suggesting its potential to be developed as a diagnostic marker [67]. It has been reported that the activation of CD44 by its ligand promotes cancer stem cell-like phenotypes in GBM and increased therapeutic resistance [68]. Consistent with this, drugs targeting CD44 are currently in clinical trials, and so far the results are promising in that CD44 inhibition impede GBM cells growth [69]. Our co-expression network analysis using graph-based analytics [15] demonstrated that genes connected to CD44 were also highly co-expressed in GBM compared to normal brain tissues, suggesting that the CD44 signalling axis is important in GBM tumorigenesis.

The currently approved therapies to treat GBM are far from satisfactory and have remained unchanged for more than a decade [70]. This includes the alkylating agent temozolomide, which is the first line of drug used in treating GBM. Therefore, there is a need for novel or alternative treatment strategies for GBM. Due to the upregulated expression of CD44 in GBM, drugs targeting CD44 are currently undergoing clinical trials and the results are thus far promising in that CD44 inhibition impedes GBM cells growth [69]. In addition to this, our drug mapping analysis revealed hyaluronic acid as an actionable CD44 binding molecule. It is therefore appealing to investigate the activity and potential use of this existing drug to treat GBM in the future, which has yet to be comprehensively studied. Within the CD44 co-expressed interactome, three additional targets already have drugs that can modulate them namely the C1R, CALR and TNFSR1A (Table 2). Based on our knowledge, the activity and efficacy of these drugs have not been tested in any in-vitro or in-vivo GBM models yet. Also, studying a combination of these available drugs targeting our GBM signature or the CD44 co-expression network could disrupt the aberrant hub gene interactome and potentially enhance GBM treatment efficacy.

## Conclusions

In summary, we identified GBM surfaceome by combining RNA-seq data. Through an integrative multi-OMICS strategy, we highlighted 6 GBM surface-enriched genes that could be important in driving GBM development. Some of these genes can be targeted by clinically approved drugs for other diseases suggesting potential drug repurposing. Additionally, further studies of these genes could lead to potential GBM diagnostic/prognostic markers or a therapeutic regimen to treat GBM.

## Supplementary Information

**Additional file 1: Supplementary Table S1.** Overall differentially expressed genes in TCGA GBM tissues vs. GTEx normal brain tissues. **Supplementary Table S2.** Significantly dysregulated cell surface genes in TCGA GBM tissues vs. GTEx normal brain tissues. **Supplementary Table S3.** GBM cell lines proteomics data from Bausch-Fluck et al. 2015. **Supplementary Table S4.** GBM tissue samples proteomics data from Polisetty et al. 2012. **Supplementary Table S5.** Protein-protein interaction network analysis of surfaceome.

**Additional file 2 Supplementary Fig. S1.** Gene ontology and deregulated pathways in GBM. (A-B) Gene ontology cellular component of the significantly (A) upregulated and (B) downregulated genes in GBM. (C-D) KEGG pathway analysis of the (C) upregulated and (D) downregulated genes in GBM. **Supplementary Fig. S2.** Significant differentially expressed cell-surface genes in GBM. (A) GBM surfaceome classification using previously annotated cell surface genes dataset identifies 395 DEGs that belongs to surfaceome. (B) Cell surface genes stratification from (A) based on its subclass. **Supplementary Fig. S3.** KEGG pathway analysis of differentially expressed surfaceome in GBM. (A) Upregulated surfaceome and (B) Downregulated surfaceome. **Supplementary Fig. S4.** Mapping the expression of 87-gene modules from (Fig. 4A) with scRNA-seq data from [26] on the basis of GBM cell microenvironment. **Supplementary Fig. S5.** Significant upregulation of the prioritized GBM surfaceome signature in GBM patients. (A-F) Boxplot showing the RNA-Seq data (transcript per million) of (A) CD44 (B) PTPRJ (C) SLC1A5 (D) EGFR (E) HLA-DRA and (F) ITGB2 in GBM and GTEx normal brain tissue samples. **Supplementary Fig. S6.** Overall survival analysis of the prioritized GBM surfaceome signature as potential GBM prognostic biomarker. (A-F) Overall survival analysis of GBM patients having high and low expression of (A) CD44 (B) PTPRJ (C) SLC1A5 (D) EGFR (E) HLA-DRA and (F) ITGB2. **Supplementary Fig. S7.** Disease-free survival analysis of the prioritized GBM surfaceome signature as potential GBM prognostic biomarker. (A-F) Disease-free survival analysis of GBM patients having high and low expression of (A) CD44 (B) PTPRJ (C) SLC1A5 (D) EGFR (E) HLA-DRA and (F) ITGB2. **Supplementary Fig. S8**. Survival analysis of the 6 GBM signature genes. (A) Overall survival and (B) disease-free survival analysis of GBM patients having high and low expression of all 6 genes; CD44, PTPRJ, SLC1A5, EGFR, HLA-DRA and ITGB2. **Supplementary Fig. S9.** Survival analysis of the 3 GBM signature genes. (A) Overall survival and (B) disease-free survival analysis of GBM patients having high and low expression of CD44, PTPRJ and HLA-DRA.

## Data Availability

The data are included within the manuscript and in the supplementary files. The TCGA GBM data can be obtained from the Genomics Data Commons Data Portal (https://portal.gdc.cancer.gov). The normal brain tissues RNA-seq data were obtained from the GTEx Portal (https://gtexportal.org/home/datasets). Other data are available from the corresponding author upon reasonable request.

## Abbreviations

GBM: Glioblastoma multiforme
TCGA: The cancer genome atlas
GTEx: Genotype-tissue expression
DEG: Differentially expressed gene
PPI: Protein-protein interaction
t-SNE: t-distributed stochastic neighbour embedding
GO: Gene Ontology
KEGG: And Kyoto encyclopedia of genes and genomes
GEPIA: Gene expression profiling interactive analysis
MHC: Major histocompatibility complex
RTK: Receptor tyrosine kinase
GPCR: G protein-coupled receptor
PTP: Protein tyrosine kinase
scRNA-seq: Single-cell RNA-sequencing
