# Supplementary material for: Integration of RNA-Seq and proteomics data identifies glioblastoma multiforme surfaceome signature
Source: BMC Cancer. 2021 Jul 23;21:850. doi: 10.1186/s12885-021-08591-0 (PMC8306276; doi:10.1186/s12885-021-08591-0)
Supplement: Supplementary file 2 — Additional file 2 Supplementary Fig. S1. Gene ontology and deregulated pathways in GBM. (A-B) Gene ontology cellular component of the significantly (A) upregulated and (B) downregulated genes in GBM. (C-D) KEGG pathway analysis of the (C) upregulated and (D) downregulated genes in GBM. Supplementary Fig. S2. Significant differentially expressed cell-surface genes in GBM. (A) GBM surfaceome classification using previously annotated cell surface genes dataset identifies 395 DEGs that belongs to surfaceome. (B) Cell surface genes stratification from (A) based on its subclass. Supplementary Fig. S3. KEGG pathway analysis of differentially expressed surfaceome in GBM. (A) Upregulated surfaceome and (B) Downregulated surfaceome. Supplementary Fig. S4. Mapping the expression of 87-gene modules from (Fig. 4A) with scRNA-seq data from [26] on the basis of GBM cell microenvironment. Supplementary Fig. S5. Significant upregulation of the prioritized GBM surfaceome signature in GBM patients. (A-F) Boxplot showing the RNA-Seq data (transcript per million) of (A) CD44 (B) PTPRJ (C) SLC1A5 (D) EGFR (E) HLA-DRA and (F) ITGB2 in GBM and GTEx normal brain tissue samples. Supplementary Fig. S6. Overall survival analysis of the prioritized GBM surfaceome signature as potential GBM prognostic biomarker. (A-F) Overall survival analysis of GBM patients having high and low expression of (A) CD44 (B) PTPRJ (C) SLC1A5 (D) EGFR (E) HLA-DRA and (F) ITGB2. Supplementary Fig. S7. Disease-free survival analysis of the prioritized GBM surfaceome signature as potential GBM prognostic biomarker. (A-F) Disease-free survival analysis of GBM patients having high and low expression of (A) CD44 (B) PTPRJ (C) SLC1A5 (D) EGFR (E) HLA-DRA and (F) ITGB2. Supplementary Fig. S8. Survival analysis of the 6 GBM signature genes. (A) Overall survival and (B) disease-free survival analysis of GBM patients having high and low expression of all 6 genes; CD44, PTPRJ, SLC1A5, EGFR, HLA-DRA and ITGB2. Supplementary Fig. [file 12885_2021_8591_MOESM2_ESM.pdf]

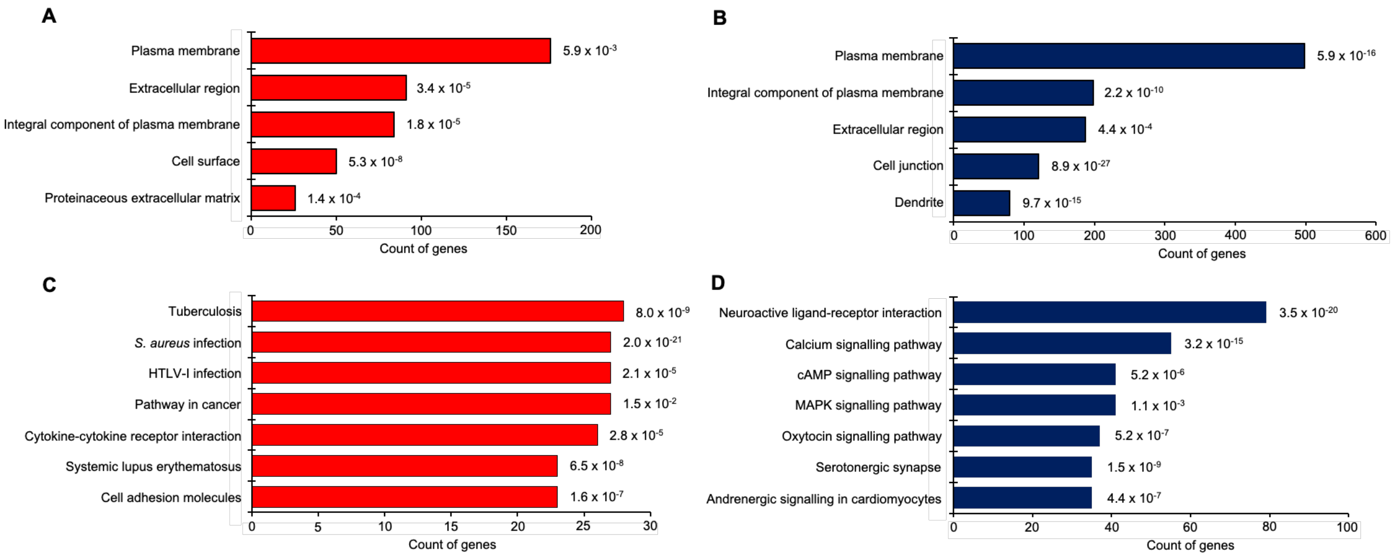

Supplementary Fig. S1. Gene ontology and deregulated pathways in GBM. (A-B) Gene ontology cellular component of the significantly (A) upregulated and (B) downregulated genes in GBM. (C-D) KEGG pathway analysis of the (C) upregulated and (D) downregulated genes in GBM.

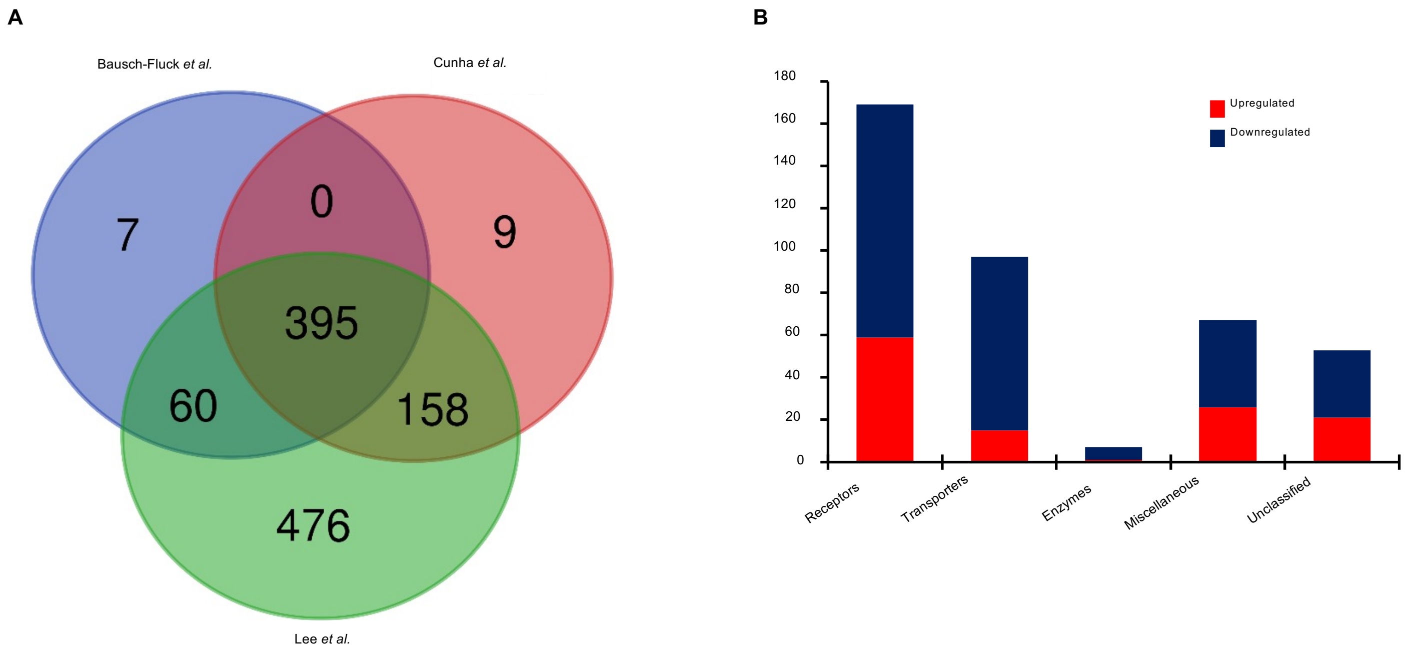

Supplementary Fig. S2. Significant differentially expressed cell-surface genes in GBM. (A) GBM surfaceome classification using previously annotated cell surface genes dataset identifies 395 DEGs that belongs to surfaceome. (B) Cell surface genes stratification from (A) based on its subclass.

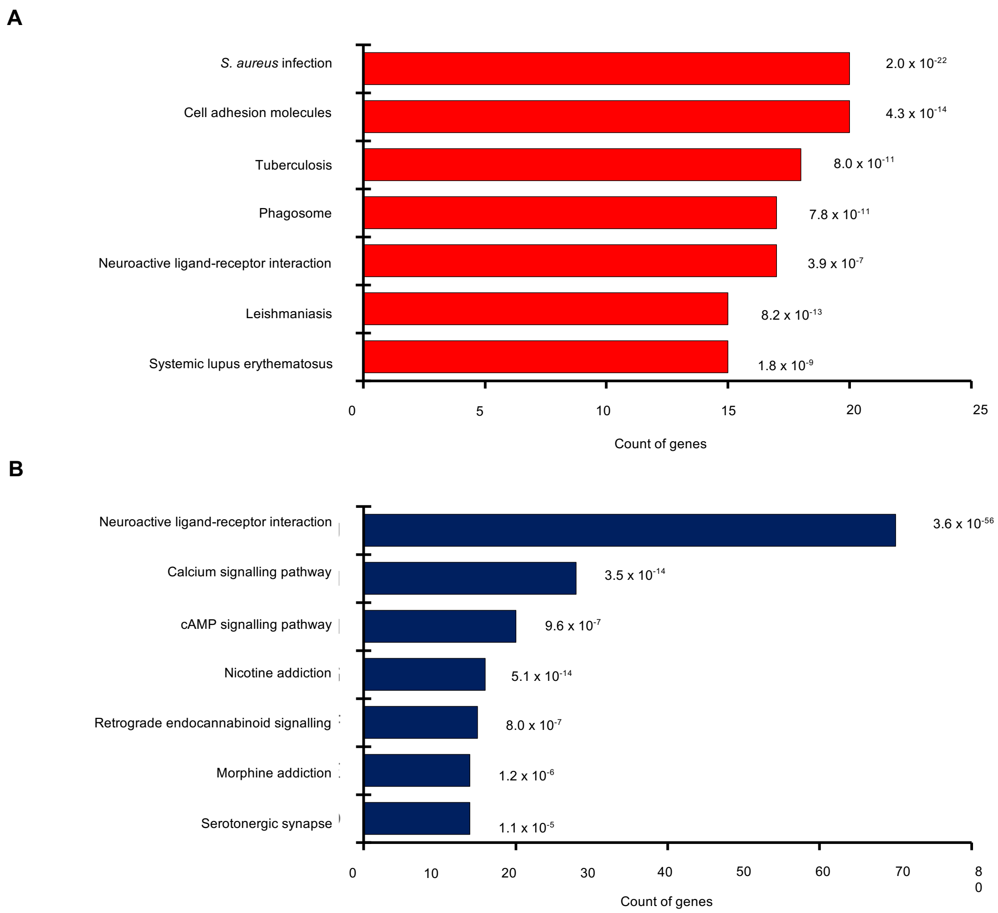

Supplementary Fig. S3. KEGG pathway analysis of differentially expressed surfaceome in GBM. (A) Upregulated surfaceome and (B) Downregulated surfaceome.

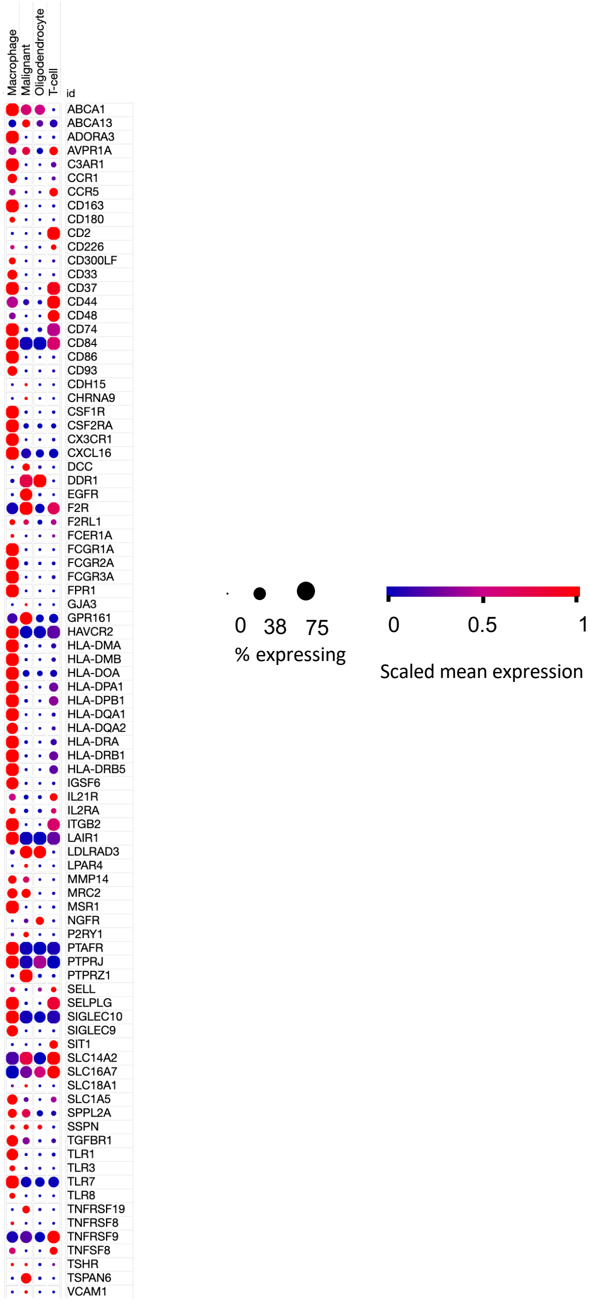

Supplementary Fig. S4. Mapping the expression of 87-gene modules from (Fig. 4A) with scRNA-seq data from [26] on the basis of GBM cell microenvironment.

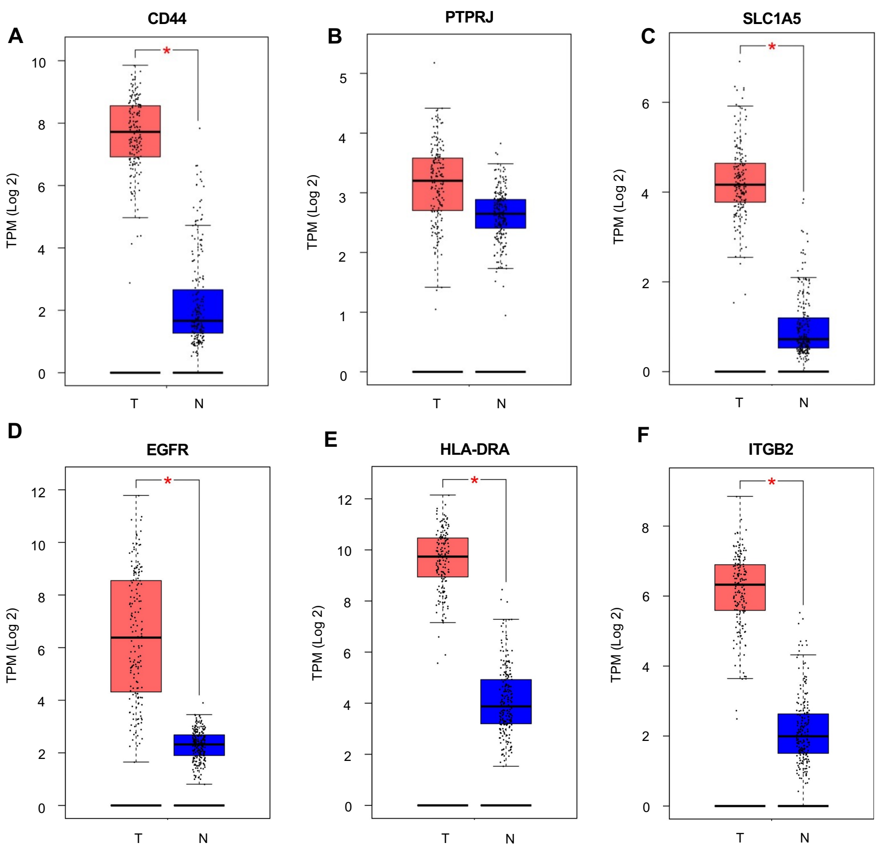

Supplementary Fig. S5. Significant upregulation of the prioritized GBM surfaceome signature in GBM patients. (A-F) Boxplot showing the RNA-Seq data (transcript per million) of (A) CD44 (B) PTPRJ (C) SLC1A5 (D) EGFR (E) HLA-DRA and (F) ITGB2 in GBM and GTEx normal brain tissue samples.

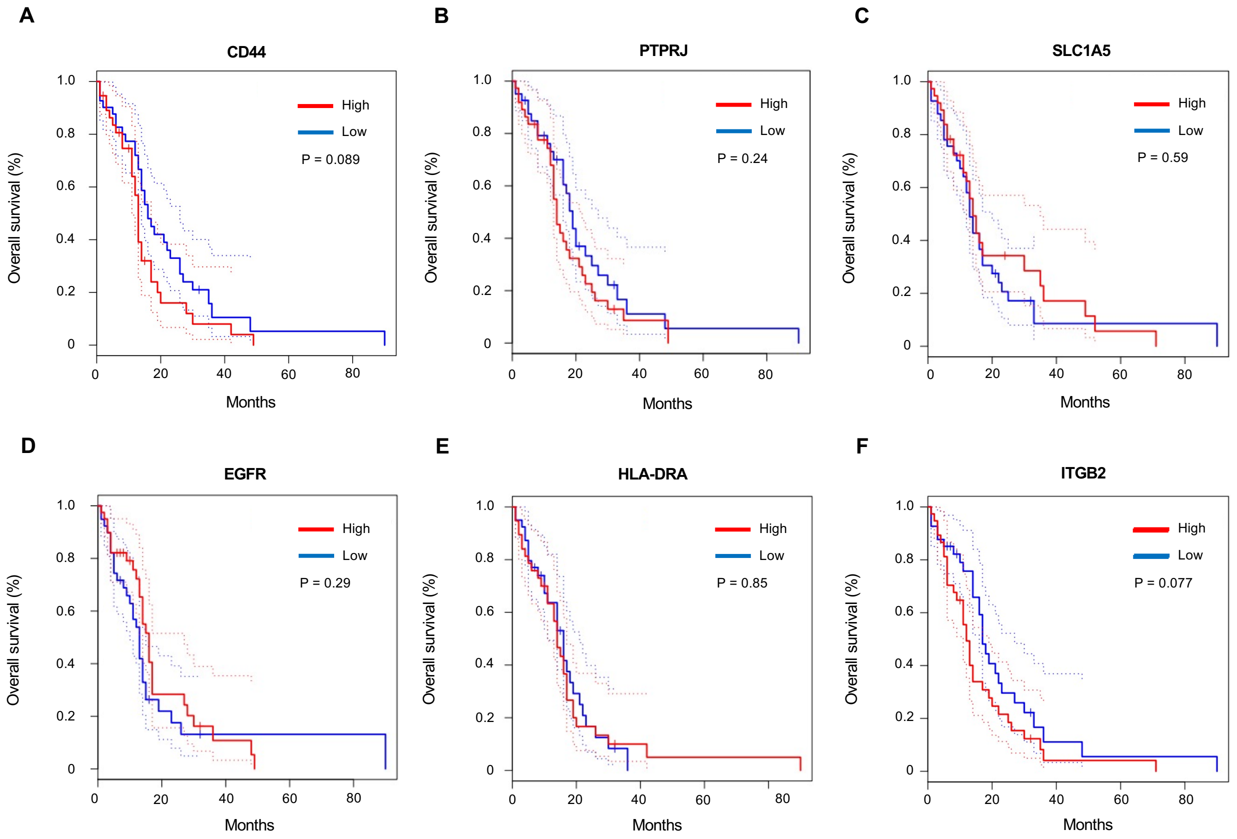

Supplementary Fig. S6. Overall survival analysis of the prioritized GBM surfaceome signature as potential GBM prognostic biomarker. (A-F) Overall survival analysis of GBM patients having high and low expression of (A) CD44 (B) PTPRJ (C) SLC1A5 (D) EGFR (E) HLA-DRA and (F) ITGB2.

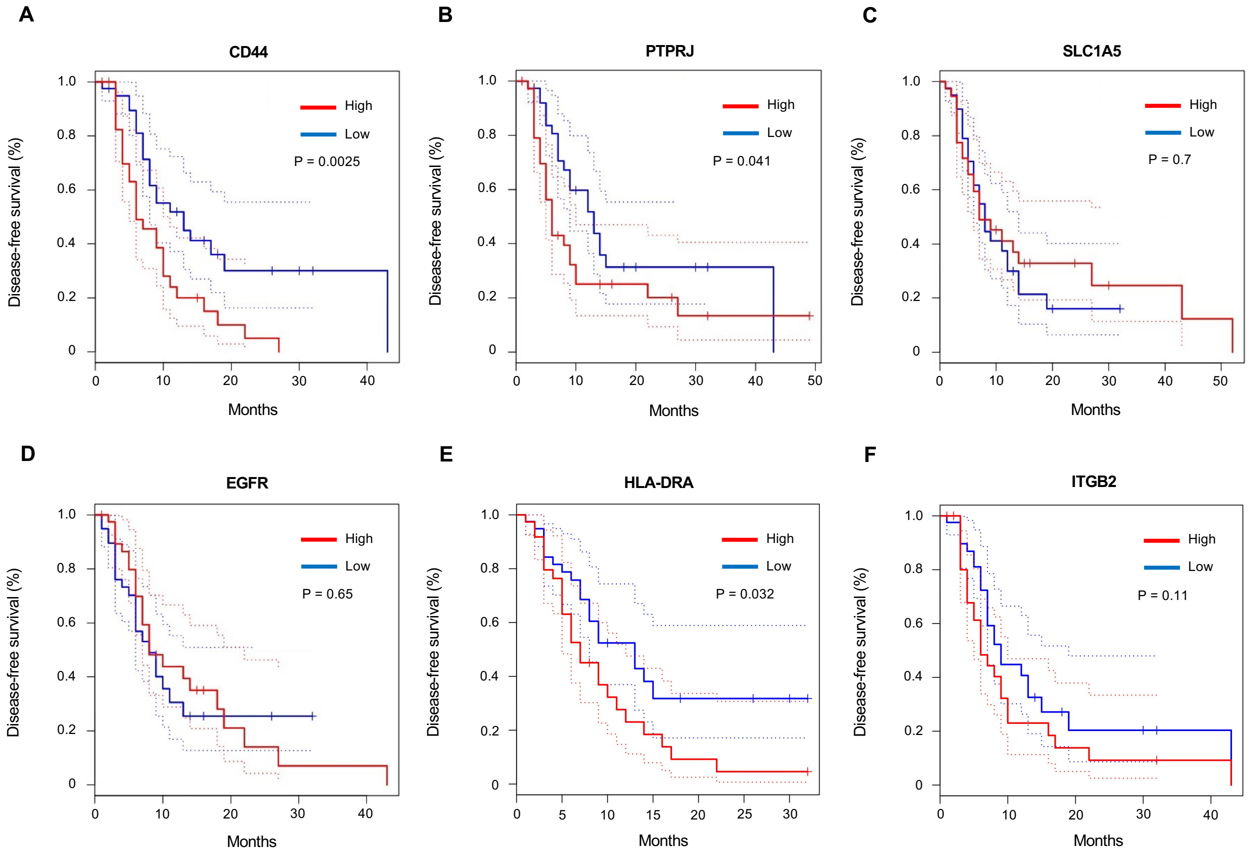

Supplementary Fig. S7. Disease-free survival analysis of the prioritized GBM surfaceome signature as potential GBM prognostic biomarker. (A-F) Disease-free survival analysis of GBM patients having high and low expression of (A) CD44 (B) PTPRJ (C) SLC1A5 (D) EGFR (E) HLA-DRA and (F) ITGB2.

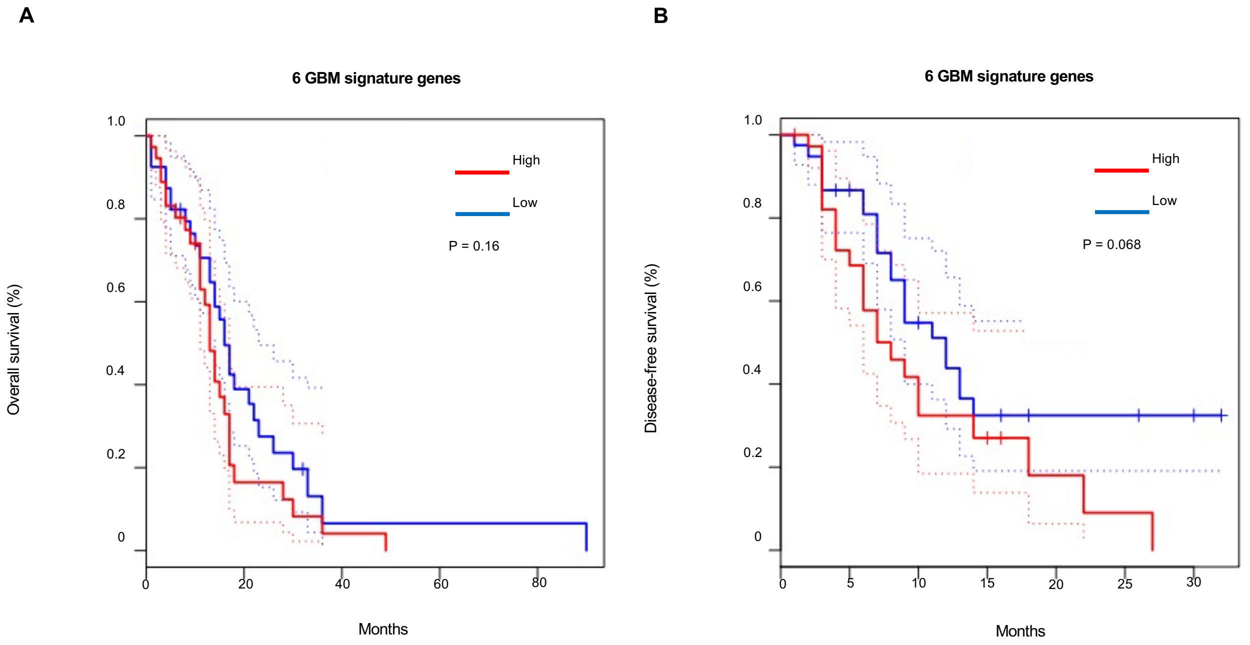

Supplementary Fig. S8. Survival analysis of the 6 GBM signature genes. (A) Overall survival and (B) disease-free survival analysis of GBM patients having high and low expression of all 6 genes; CD44, PTPRJ, SLC1A5, EGFR, HLA-DRA and ITGB2.

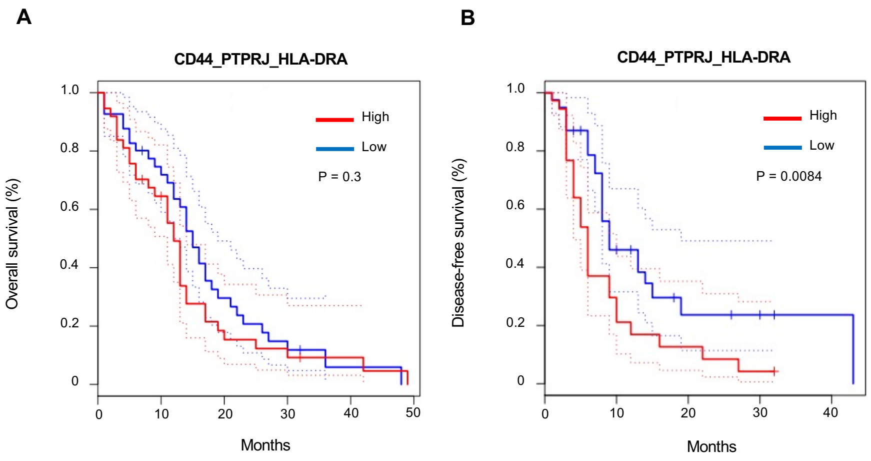

Supplementary Fig. S9. Survival analysis of the 3 GBM signature genes. (A) Overall survival and (B) disease-free survival analysis of GBM patients having high and low expression of CD44, PTPRJ and HLA-DRA.
